# Efficacy of revefenacin, a long-acting muscarinic antagonist for nebulized therapy, in patients with markers of more severe COPD: a post hoc subgroup analysis

**DOI:** 10.1186/s12890-020-1156-4

**Published:** 2020-05-11

**Authors:** James F. Donohue, Edward Kerwin, Chris N. Barnes, Edmund J. Moran, Brett Haumann, Glenn D. Crater

**Affiliations:** 1 Pulmonary Medicine, UNC School of Medicine, 321 S Columbia St, Chapel Hill, NC 27516 USA; 2 Clinical Research Institute of Southern Oregon, LLC, 3860 Crater Lake Ave, Medford, OR 97504 USA; 3 Theravance Biopharma US, Inc., 901 Gateway Blvd, South San Francisco, CA 94080 USA

**Keywords:** COPD, Efficacy, Long-acting muscarinic antagonist, Nebulized therapy, Revefenacin

## Abstract

**Background:**

Revefenacin, a once-daily, long-acting muscarinic antagonist delivered via standard jet nebulizer, increased trough forced expiratory volume in 1 s (FEV_1_) in patients with moderate to very severe chronic obstructive pulmonary disease (COPD) in prior phase 3 trials. We evaluated the efficacy of revefenacin in patients with markers of more severe COPD.

**Methods:**

A post hoc subgroup analysis of two replicate, randomized, phase 3 trials was conducted over 12 weeks. Endpoints included least squares change from baseline in trough FEV_1_, St. George’s Respiratory Questionnaire (SGRQ) responders, and transition dyspnea index (TDI) responders at Day 85. This analysis included patient subgroups at high risk for COPD exacerbations and compared patients who received revefenacin 175 μg and placebo: severe and very severe airflow limitation (percent predicted FEV_1_ 30%–< 50% and < 30%), 2011 Global Initiative for Chronic Obstructive Lung Disease (GOLD) D, reversibility (≥ 12% and ≥ 200 mL increase in FEV_1_) to short-acting bronchodilators, concurrent use of long-acting β agonists and/or inhaled corticosteroids, older age (> 65 and > 75 years), and comorbidity risk factors.

**Results:**

Revefenacin demonstrated significant improvements in FEV_1_ versus placebo at Day 85 among the intention-to-treat (ITT) population and all subgroups. Additionally, there was a greater number of SGRQ and TDI responders in the ITT population and the majority of subgroups analyzed among patients who received revefenacin versus placebo. For the SGRQ responders, the odds of response (odds ratio > 2.0) were significantly greater in the revefenacin arm versus the placebo arm among the severe airflow obstruction, very severe airflow obstruction and 2011 GOLD D subgroups. For the TDI responders, the odds of response (odds ratio > 2.0) were significantly greater among the severe airflow obstruction subgroup and patients aged > 75 years.

**Conclusions:**

Revefenacin showed significantly greater improvements in FEV_1_ versus placebo in the ITT population and all subgroups. Furthermore, there were a greater number of SGRQ and TDI responders in the ITT population, and in the majority of patient subgroups among patients who received revefenacin versus placebo. Based on the data presented, revefenacin could be a therapeutic option among patients with markers of more severe COPD.

**Trial registration:**

Clinical trials registered with www.clinicaltrials.gov (Studies 0126 [NCT02459080; prospectively registered 22 May 2015] and 0127 [NCT02512510; prospectively registered 28 July 2015]).

## Background

Inhaled drug delivery is the foundation of chronic obstructive pulmonary disease (COPD) pharmacological treatment [1]. The most common devices used to administer aerosolized medication in day-to-day respiratory practice are the pressurized metered-dose inhaler (MDIs) and dry powder inhaler (DPIs) [2]. The ability to use these inhalers adequately may become problematic among patients with COPD whose disease and symptoms become more severe. For pressurized MDIs, patients need to inhale correctly and coordinate breathing and actuation to ensure effective drug delivery. For DPIs, patients may struggle to generate sufficient inspiratory capacity to overcome the internal resistance of the device to de-aggregate the powdered drug into fine particles small enough for lung deposition [2, 3].

The Global Initiative for Chronic Obstructive Lung Disease (GOLD) report recognize that markers (eg, symptoms and exacerbations), other than lung function impairment are associated with more severe disease [1]. Nebulized therapy may be an option in patients with more severe markers of COPD. Nebulized bronchodilators are recommended for patients with COPD who have very low inspiratory flow rates, physical, or mental impairments that preclude the use of inhalers, including elderly patients and patients with severe disease. They are also available to patients with COPD who prefer nebulized therapies [2, 4, 5].

Revefenacin inhalation solution is a once-daily long-acting muscarinic antagonist delivered by a standard jet nebulizer that is approved by the US Food and Drug Administration (FDA) for the maintenance treatment of patients with COPD [6]. The efficacy of revefenacin has been demonstrated in previous randomized, controlled, phase 3 trials in broad populations of patients with moderate to very severe COPD with or without concurrent long-acting ß agonist (LABA). Revefenacin significantly improved lung function (trough forced expiratory volume in 1 s [FEV_1_] and overall treatment effect FEV_1_) compared with placebo in two replicate 12-week studies [7]. Revefenacin treatment was shown to improve FEV_1_ and respiratory health outcomes in a 52-week study with results similar to tiotropium via HandiHaler® [8]. Revefenacin was well tolerated for 52 weeks and has a safety profile that supports its long-term use in patients with COPD [9]. In addition, revefenacin was not associated with adverse cardiovascular events [10, 11]. Therefore, it may provide a beneficial treatment option for patients with cardiovascular disease, one of the most common comorbidities among patients with COPD [12].

Identifying patient subgroups who are most likely to benefit from nebulized long-acting muscarinic antagonist (LAMA) treatment can help clinicians direct therapy to patients at high risk for COPD exacerbations. Here, in this post hoc subgroup analysis, we evaluated the efficacy, and health outcomes of revefenacin 175 μg versus placebo, in patients with markers of more severe COPD who participated in the replicate, placebo-controlled, 12-week phase 3 trials (0126 and 0127). Some of the methods and results of this analysis were previously reported in the form of an abstract [13].

## Methods

### Study design and conduct

Studies 0126 (NCT02459080) and 0127 (NCT02512510) were replicate, 12-week, randomized, double-blind, placebo-controlled, multiple-dose, parallel-group, phase 3 trials, and the design and conduct were described previously [7]. The studies were approved conducted according to the principles of the International Council on Harmonisation of Technical Requirements for Pharmaceuticals for Human Use guideline for good clinical practice [14], and the code of ethics of the World Medical Association’s Declaration of Helsinki [15]; written informed consent was obtained from all patients. The protocol was reviewed and approved by an institutional review board (Quorum Review IRB, Seattle, Washington).

### Patients

Inclusion and exclusion criteria have been described previously [7]. Briefly, patients aged ≥ 40 years with moderate to very severe COPD, a smoking history ≥ 10 pack-years, post-ipratropium FEV_1_/forced vital capacity ratio < 0.7, and post-ipratropium FEV_1_ < 80% of predicted normal and > 700 mL at screening were enrolled. Concomitant LABAs (with or without inhaled corticosteroids [ICS]) was permitted in up to 40% of the study population to ensure robust assessments of concurrent therapies used by patients. Once the 40% cap was reached, new patients who entered screening required a 14-day washout of any LABA-containing therapy before the ipratropium reversibility test at screening. Patients taking ICS/LABA at enrollment were switched to receive ICS monotherapy at an equivalent dose for at least 14 days, before the ipratropium reversibility visit at screening. Stable doses of ICS without concomitant LABAs were permitted, but LAMAs and short-acting muscarinic antagonists were prohibited.

Patients were randomized (1:1:1) in a double-blind manner to receive revefenacin 175 μg, revefenacin 88 μg, or placebo once daily via PARI LC® Sprint (Starnberg, Germany) jet nebulizer for 12 weeks. Results with revefenacin 175 μg, which is the FDA-approved dose, are reported here.

### Analysis population and endpoints

Endpoints of this study included the least squares (LS) change from baseline in trough FEV_1_ at Day 85, St. George’s Respiratory Questionnaire (SGRQ) responders, and transition dyspnea index (TDI) responders at Day 85. This analysis included the intention-to-treat (ITT) population and subgroups of patients at high risk for COPD exacerbations, and compared patients who received revefenacin 175 μg and placebo. The following subgroups of patients were analyzed: severe airflow limitation (percent predicted FEV_1_ 30%–< 50%), very severe airflow limitation (percent predicted FEV_1_ < 30%), 2011 GOLD D, patients that are reversible (≥ 12% and ≥ 200 mL increase in FEV_1_) to short-acting bronchodilators (ipratropium and albuterol), background ICS, background LABA and/or ICS, older age (defined as > 65 or > 75 years), and comorbidity risk factors which included history of cardiovascular disease, diabetes mellitus, and cognitive/mental impairments.

### Statistical analyses

The full analysis set included all randomized patients who received at least one dose of study drug and had at least one recorded post-baseline FEV_1_ assessment. Pooled analyses were conducted using a repeated statement of subject ID nested within study instead of a random statement to ensure convergence. Changes from baseline in FEV_1_ were analyzed using a mixed model for repeated measures. Trough FEV_1_ at Day 85 is defined as the mean of the 23.25- and 23.75-h spirometry assessments post the Day 84 dose. Trough FEV_1_ at Days 15, 29, 57, and 84 is defined as the mean of the − 45 min and − 15 min pre-dose spirometry assessments. SGRQ and TDI responders were the proportions of patients with a reduction in SGRQ total score ≥ 4 units, or an increase in TDI score ≥ 1 unit (ie, minimum clinically important differences [MCID]), respectively [16, 17].

## Results

### Patient demographics and baseline characteristics

Data from 812 patients were pooled for analysis, with 395 patients receiving revefenacin 175 μg, and 417 patients receiving placebo (Table 1). Across both treatment groups, approximately 45% of patients were > 65 years, 10% were > 75 years, and 37% were on background LABA and/or ICS. In addition, approximately 31% of patients had severe airflow limitation (percent predicted FEV_1_ 30%–< 50%), and 34% met 2011 GOLD D criteria. For comorbidities, approximately 47%, 20%, and 15% of patients had a history of cardiovascular disease, diabetes mellitus, and cognitive/mental impairments, respectively. Overall, patient demographics and baseline characteristics from the pooled analysis indicated that revefenacin and placebo groups were well balanced across all variables (Table 1).

**Table 1:** Pooled population demographics and baseline characteristics

Characteristic	Revefenacin 175 μg (*n* = 395)	Placebo (*n* = 417)
Sex, male, *n* (%)	195 (49.4)	206 (49.4)
> 65 years, *n* (%)	176 (44.6)	185 (44.4)
> 75 years, *n* (%)	35 (8.9)	42 (10.1)
Current smoker, *n* (%)	190 (48.1)	198 (47.5)
Concurrent LABA or ICS/LABA, *n* (%)	153 (38.7)	147 (35.3)
Concurrent ICS, *n* (%)	174 (44.1)	171 (41.0)
FEV_1_ 30%–< 50% pred, *n* (%)	119 (30.1)	134 (32.1)
FEV_1_ < 30% pred, *n* (%)	26 (6.6)	16 (3.8)
2011 GOLD category D, *n* (%)	132 (33.4)	141 (33.8)
Reversible to ipratropium and albuterol, *n* (%)	86 (21.8)	82 (19.7)
History of cardiovascular disease^a^	178 (45.1)	200 (48.0)
History of diabetes	80 (20.3)	78 (18.7)
History of cognitive/mental impairments	58 (14.7)	61 (14.6)

^a^Cardiovascular risk factors: aged ≥ 60 years and any two of the following conditions: diabetes, hypercholesterolemia, hypertension, peripheral vascular disorder or cardiac disorders from reported medical history or aged ≥ 40 years and a cardiac disorder(s) from reported medical history

*FEV*_*1*_ forced expiratory volume in 1 s; *GOLD* Global Initiative for Chronic Obstructive Lung Disease; *ICS* inhaled corticosteroids; *LABA* long-acting ß agonist

### Changes from baseline in trough FEV_1_

**Fig. 1:**
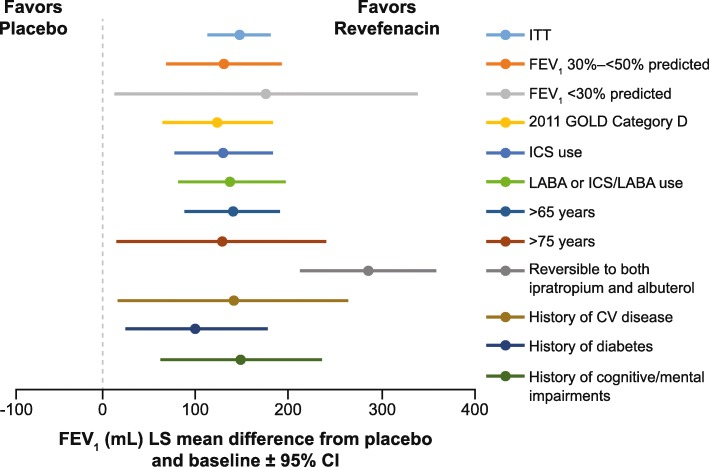
Day 85 trough FEV_1_ by patient subgroup. The LS mean difference for revefenacin versus placebo was statistically significant (*p* < 0.05) for all subgroups. *CI* confidence intervals; *CV* cardiovascular; *FEV*_*1*_ forced expiratory volume in 1 s; *GOLD* Global Initiative for Chronic Obstructive Lung Disease; *LABA* long-acting ß agonist; *ICS* inhaled corticosteroids; *ITT* intention-to-treat

Across the ITT population and subgroups, revefenacin 175 μg produced significantly greater improvements in Day 85 trough FEV_1_ than placebo (Fig. 1 and Table 2). Of note, revefenacin demonstrated significantly greater improvements in trough FEV_1_ among patients who are reversible to short-acting bronchodilators versus placebo (LS mean [95% confidence intervals] difference, 286.52 [214.8, 358.2] mL, *p* < 0.0001), In addition, revefenacin demonstrated significant increases in FEV_1_ among elderly patients (aged > 75 years, and > 65 years), providing additional 129–140 mL improvements versus placebo (both *p*-values < 0.03). Among patients with comorbidities, revefenacin demonstrated significantly greater improvements in trough FEV_1_ among patients with a history of diabetes mellitus, cardiovascular disease, and cognitive/mental impairments, providing additional 102–150 mL improvements versus placebo (all *p*-values < 0.03) (Fig. 1 and Table 2).

**Table 2:** Day 85 trough FEV_1_ (mL) by patient subgroup

Subgroups	Revefenacin 175 μg (*n* = 395)	Placebo (*n* = 417)
**ITT**		
Evaluable n	310	296
LS mean difference (95% CI)	148.1 (115.2, 181.1); *p* < 0.0001	
**FEV**_**1**_**30%–< 50% pred**		
Evaluable n	101	78
LS mean difference (95% CI)	131.2 (70.7, 191.6), *p* < 0.0001	
**FEV**_**1**_ **< 30% pred**		
Evaluable n	17	9
LS mean difference (95% CI)	176.2 (14.7, 337.5), *p* = 0.0324	
**2011 GOLD category D**		
Evaluable n	108	83
LS mean difference (95% CI)	124.6 (66.5, 182.7), *p* < 0.0001	
**ICS use**		
Evaluable n	135	108
LS mean difference (95% CI)	130.6 (78.7, 182.5), *p* < 0.001	
**LABA or ICS/LABA use**		
Evaluable n	118	89
LS mean difference (95% CI)	139.2 (82.9, 195.5), *p* < 0.0001	
**> 65 years**		
Evaluable n	143	128
LS mean difference (95% CI)	140.3 (91.0, 189.7), *p* < 0.0001	
**> 75 years**		
Evaluable n	28	25
LS mean difference (95% CI)	129.2 (18.9, 239.5), *p* = 0.0217	
**Reversible to ipratropium and albuterol**		
Evaluable n	70	57
LS mean difference (95% CI)	286.5 (214.8, 358.2), *p* < 0.0001	
**History of CV disease**		
Evaluable n	21	27
LS mean difference (95% CI)	140.7 (18.4, 263.0), *p* = 0.0242	
**History of diabetes**		
Evaluable n	85	57
LS mean difference (95% CI)	101.6 (27.0, 176.3), *p* = 0.0077	
**History of cognitive/mental impairments**		
Evaluable n	45	44
LS mean difference (95% CI)	149.5 (64.5, 234.5), *p* = 0.0006	

*CI* confidence intervals; *CV* cardiovascular; *FEV*_*1*_ forced expiratory volume in 1 s; *GOLD* Global Initiative for Chronic Obstructive Lung Disease, *LABA* long-acting ß agonist; *ICS* inhaled corticosteroids; *ITT* intention-to-treat; *pred* predicted

### SGRQ responders

**Fig. 2:**
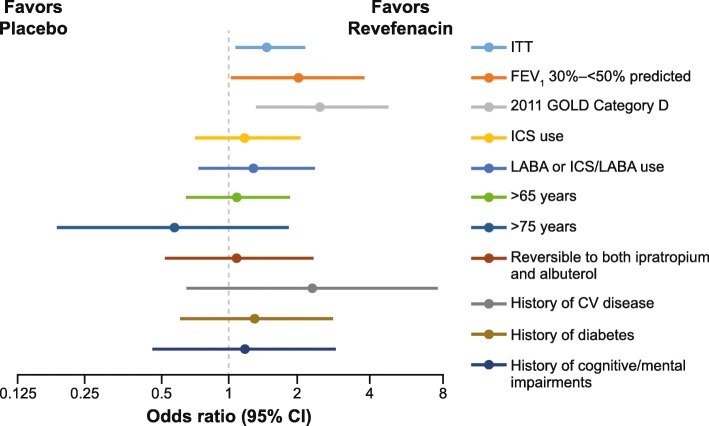
Day 85 SGRQ responders by patient subgroup. The odds ratios for revefenacin versus placebo was statistically significant (*p* < 0.05) for the following subgroups: ITT, FEV_1_ 30%–< 50% predicted and, 2011 GOLD category D. The subgroup, FEV_1_ < 30% predicted, has been excluded from the forest plot due to being out with the range of the x-axis scale. *CI* confidence intervals; *CV* cardiovascular; *FEV*_*1*_ forced expiratory volume in 1 s; *GOLD* Global Initiative for Chronic Obstructive Lung Disease; *LABA* long-acting ß agonist; *ICS* inhaled corticosteroids; *ITT* intention-to-treat; *SGRQ* St. George’s Respiratory Questionnaire

In the ITT population, a higher proportion of patients in the revefenacin 175 μg arm (46.9%) met the MCID criteria of SGRQ responder than placebo (36.2%) (Fig. 2 and Table 3), with the odds of response significantly greater in the revefenacin 175 μg arm than in the placebo arm (*p* = 0.0116). In general, the majority of subgroup analyses showed a higher rate of responders for revefenacin than for placebo, with the odds of response (odds ratio > 2.0) significantly greater in the revefenacin arm than in the placebo arm, among the severe (*p* = 0.037) and very severe (*p* < 0.001) airflow limitations, and 2011 GOLD D (*p* = 0.004) subgroups. In addition, the cardiovascular disease subgroup showed a non-significant trend, with the odds of response exceeding 2.0; odds ratio 2.3 (95% confidence intervals 0.68–7.83, *p* = 0.1822) (Fig. 2 and Table 3).

**Table 3:** Day 85 SGRQ responders by patient subgroup

Subgroups	Revefenacin 175 μg (*n* = 395)	Placebo (*n* = 417)
**ITT**		
Evaluable n	288	276
Odds ratio (95% CI)	1.53 (1.10, 2.13), *p* = 0.0116	
**FEV**_**1**_**30%–< 50% pred**		
Evaluable n	96	78
Odds ratio (95% CI)	1.99 (1.04, 3.81), *p* = 0.0368	
**FEV**_**1**_ **< 30% pred**		
Evaluable n	16	7
Odds ratio (95% CI)	2 × 10^10^ (3.05 × 10^7^,126 × 10^9^), *p* < 0.001	
**2011 GOLD category D**		
Evaluable n	103	81
Odds ratio (95% CI)	2.52 (1.34, 4.76), *p* = 0.0042	
**ICS use**		
Evaluable n	134	105
Odds ratio (95% CI)	1.23 (0.74, 2.03), *p* = 0.4291	
**LABA or ICS/LABA use**		
Evaluable n	118	85
Odds ratio (95% CI)	1.34 (0.77, 2.35), *p* = 0.2995	
**> 65 years**		
Evaluable n	133	119
Odds ratio (95% CI)	1.11 (0.67, 1.84), *p* = 0.6897	
**> 75 years**		
Evaluable n	28	25
Odds ratio (95% CI)	0.58 (0.19, 1.81), *p* = 0.3506	
**Reversible to ipratropium and albuterol**		
Evaluable n	66	51
Odds ratio (95% CI)	1.12 (0.55, 2.30), *p* = 0.7486	
**History of CV disease**		
Evaluable n	19	25
Odds ratio (95% CI)	2.30 (0.68, 7.83), *p* = 0.1822	
**History of diabetes**		
Evaluable n	60	53
Odds ratio (95% CI)	1.31 (0.63, 2.75), *p* = 0.4704	
**History of cognitive/mental impairments**		
Evaluable n	43	40
Odds ratio (95% CI)	1.18 (0.49, 2.88), *p* = 0.7126	

*CI* confidence intervals; *CV* cardiovascular; *FEV*_*1*_ forced expiratory volume in 1 s; *GOLD* Global Initiative for Chronic Obstructive Lung Disease, *LABA* long-acting ß agonist; *ICS* inhaled corticosteroids; *ITT* intention-to-treat; *pred* predicted

### TDI responders

**Fig. 3:**
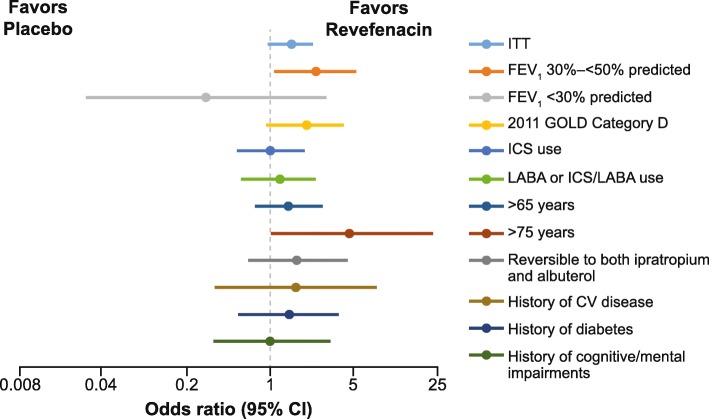
Day 85 TDI responders by patient subgroup. The odds ratios for revefenacin versus placebo was statistically significant (*p* < 0.05) for the following subgroups: FEV_1_ 30%–< 50% predicted, and > 75 years. *CI* confidence intervals; *CV* cardiovascular; *FEV*_*1*_ forced expiratory volume in 1 s; *GOLD* Global Initiative for Chronic Obstructive Lung Disease; *LABA* long-acting ß agonist; *ICS* inhaled corticosteroids; *ITT* intention-to-treat; *TDI* transition dyspnea index

In the ITT population, a higher proportion of patients in the revefenacin arm (55.0%) met the MCID criteria of TDI responder than placebo (47.2%), with the odds of response greater in the revefenacin arm than in the placebo arm (Fig. 3 and Table 4). Overall, the majority of subgroup analyses showed a higher rate of responders for revefenacin than for placebo, with the odds of response significantly greater among the severe airflow obstruction subgroup, odds ratio 2.37 (95% confidence intervals 1.10–5.08, *p* = 0.027), and a tendency towards significance in the 2011 GOLD D subgroup, odds ratio 1.95 (95% confidence intervals 0.93–4.09, *p* = 0.079). In addition, the odds of being a TDI responder were significantly greater in the revefenacin arm than in the placebo among patients aged > 75 years; odds ratio 4.7 (95% confidence intervals 1.02–21.86, *p* = 0.047) (Fig. 3 and Table 4).

**Table 4:** Day 85 TDI responders by patient subgroup

Subgroups	Revefenacin 175 μg (*n* = 395)	Placebo (*n* = 417)
**ITT**		
Evaluable n	280	271
Odds ratio (95% CI)	1.46 (0.96, 2.22), *p* = 0.0760	
**FEV**_**1**_**30%–< 50% pred**		
Evaluable n	95	77
Odds ratio (95% CI)	2.37 (1.10, 5.08), *p* = 0.0268	
**FEV**_**1**_ **< 30% pred**		
Evaluable n	15	7
Odds ratio (95% CI)	0.31 (0.03, 2.88), *p* = 0.3016	
**2011 GOLD category D**		
Evaluable n	101	80
Odds ratio (95% CI)	1.95 (0.93, 4.09), *p* = 0.0789	
**ICS use**		
Evaluable n	131	100
Odds ratio (95% CI)	1.04 (0.54, 1.98), *p* = 0.9115	
**LABA or ICS/LABA use**		
Evaluable n	116	81
Odds ratio (95% CI)	1.16 (0.57, 2.35), *p* = 0.6845	
**> 65 years**		
Evaluable n	131	115
Odds ratio (95% CI)	1.43 (0.76, 2.68), *p* = 0.2687	
**> 75 years**		
Evaluable n	28	25
Odds ratio (95% CI)	4.72 (1.02, 21.86), *p* = 0.0470	
**Reversible to ipratropium and albuterol**		
Evaluable n	63	50
Odds ratio (95% CI)	1.72 (0.67, 4.38), *p* = 0.2583	
**History of CV disease**		
Evaluable n	18	25
Odds ratio (95% CI)	1.62 (0.93, 2.22), *p* = 0.5397	
**History of diabetes**		
Evaluable n	61	51
Odds ratio (95% CI)	1.41 (0.55, 3.64), *p* = 0.4719	
**History of cognitive/mental impairments**		
Evaluable n	41	38
Odds ratio (95% CI)	1.03 (0.34, 3.10), *p* = 0.9552	

*CI* confidence intervals; *CV* cardiovascular; *FEV*_*1*_ forced expiratory volume in 1 s; *GOLD* Global Initiative for Chronic Obstructive Lung Disease, *LABA* long-acting ß agonist; *ICS* inhaled corticosteroids; *ITT* intention-to-treat; *pred* predicted

## Discussion

This post hoc subgroup analysis of two replicate, randomized, double-blind, placebo-controlled, parallel-group, 12-week phase 3 trials (0126 and 0127) provides evidence for the efficacy of revefenacin delivered by a standard jet nebulizer in patients with COPD that had markers of severe disease. This analysis of pooled data from Studies 0126 and 0127 in all subgroups of patients with COPD that had markers of severe disease, showed that revefenacin was associated with significant improvements in lung function (range, 102–176 mL), which was comparable with the ITT population (148 mL).

In addition, revefenacin demonstrated improvements in health-related quality of life (as measured by SGRQ responders) and dyspnea (as measured by TDI responders) in the majority of patient subgroups versus placebo; these improvements were also comparable to those observed in the ITT population. The odds of being a SGRQ responder were significantly greater among patients with severe airflow obstruction (percent predicted FEV_1_ 30%–< 50%), very severe airflow obstruction (percent predicted FEV_1_ < 30%), and those classified as 2011 GOLD D. Among patients with comorbidities, the odds of response in the revefenacin group with a history of cardiovascular disease showed a non-significant trend (odds ratio > 2.0) compared with placebo. It is likely significance was not met due to the relatively small patient numbers. The odds of being a TDI responder were significantly greater among patients with severe airflow obstruction (percent predicted FEV_1_ 30%–< 50%), and those aged > 75 years, and there was a tendency towards significance in the 2011 GOLD D subgroup.

Results of this analysis are consistent with other studies that evaluated the efficacy of patients taking revefenacin and a concomitant LABA or LABA/ICS, or combining other LAMAs with LABA or LABA/ICS. Revefenacin 175 μg demonstrated improvements in FEV_1_ in concomitant LABA patients in a 52 week study [8]. The efficacy of combined LAMA/LABA treatments has been shown to improve lung function and health outcomes [18–20]. In a systematic review and meta-analysis, it was reported that combining LAMA with LABA and ICS in patients with advanced COPD have better lung function and health-related quality of life and lower rates of moderate/severe COPD exacerbations than dual therapy or monotherapy [21].

In this analysis, revefenacin resulted in significant improvements in lung function, SGRQ and TDI among patients with severe airflow obstruction (percent predicted FEV_1_ 30%–< 50%) and classified as GOLD D in this study, which is consistent with previous studies. Nebulized glycopyrrolate was shown to improve FEV_1_, SGRQ, and TDI in patients with moderate to very severe COPD [22]. Furthermore, tiotropium demonstrated higher efficacy versus salmeterol in prolonging time to first COPD exacerbation and reducing number of exacerbations in patients both at high exacerbation risk [18]. In addition, aclidinium 400 μg significantly improved respiratory symptoms among patients who were classified as GOLD D at baseline [23].

Patients with COPD frequently have comorbid conditions, which can influence mortality and hospitalizations [1]. In this study, revefenacin demonstrated significant improvements in FEV_1_ and health outcomes among patient subgroups with cardiovascular disease, and diabetes mellitus compared with patients who received placebo. Similarly, nebulized glycopyrrolate improved FEV_1_, and patient-reported outcomes in patients with COPD, irrespective of cardiovascular risk status [24]. In previous studies of patients with COPD and comorbid type 2 diabetes, ICS therapy may have a negative impact on diabetes control, and patients prescribed higher doses may be at greater risk of diabetes progression [25, 26]. In the GOLD report, combination ICS/LABA or LAMA/LABA or LAMA monotherapy are recommended for GOLD D patients [1]. However, in patients with comorbid diabetes, it may be more appropriate to limit the use of ICS to the minority of patients with COPD who might benefit.

There were no safety issues identified with the use of revefenacin in patients with cardiac risk factors [7, 9]. In a preclinical study, revefenacin was shown to be a high-affinity competitive antagonist at human recombinant muscarinic acetylcholine receptors with kinetic functional selectivity for M_3_ over M_2_ muscarinic acetylcholine receptors [27]. In addition, revefenacin is a metabolically labile primary amide “soft-drug” site that allows rapid systemic clearance of the parent drug, thus potentially minimizing systemically mediated adverse events [27, 28].

Results of this analysis also demonstrated significant improvements in FEV_1_ in patients who received revefenacin among subgroups aged > 65 years and > 75 years, and cognitive/mental impairments, versus those who received placebo. Similarly, a retrospective analysis demonstrated the efficacy and safety of tiotropium among elderly patients with COPD (< 70 years, 70–79 years, and ≥ 80 years) [29]. Previous studies have suggested that nebulized therapy may be an appropriate option in patients with COPD and arthritis, impaired manual dexterity, chronic muscle weakness, or mental health or confusion disorders, or who are in hospitals, tertiary care centers, and assisted care settings as they may prefer nebulized therapy that is easy to use and does not require special training [2, 30].

Several limitations should be noted for this study. The treatment period was only three months, which does not allow for conclusions on long-term treatment. Due to small sample sizes in the subgroups and post hoc nature of this study, results should be interpreted with caution. The populations assessed in this study had stable COPD and did not include patients that had recent hospitalizations or respiratory infections. Peak inspiratory flow rate was not assessed at baseline, and therefore, patients with a suboptimal peak inspiratory flow rate could not be assessed as a potential population with markers of more severe COPD.

## Conclusions

In summary, in this post hoc subgroup analysis of data from Studies 0126 and 0127 among patients with markers of more severe COPD, revefenacin treatment showed significant improvements in lung function. In addition, there was a greater number of SGRQ and TDI responders in the ITT population and the majority of patient subgroups among patients who received revefenacin versus placebo. Based on the data presented, revefenacin could be a therapeutic option among patients with markers of more severe COPD.

## Data Availability

Theravance Biopharma (and its affiliates) will not be sharing individual de-identified participant data or other relevant study documents. Theravance Biopharma reviews the appropriateness of public disclosure of de-identified study data on a regular basis; however, at this time, has determined that public disclosure is not appropriate for advancing the knowledge around COPD treatment.

## Abbreviations

COPD: Chronic obstructive pulmonary disease
DPI: Dry powder inhaler
FDA: Food and Drug Administration
FEV_1_: Forced expiratory volume in 1 s
GOLD: Global Initiative for Chronic Obstructive Lung Disease
ICS: Inhaled corticosteroid
ITT: Intention-to-treat
LABA: Long-acting ß agonist
LAMA: Long-acting muscarinic antagonist
MDI: Metered-dose inhaler
SGRQ: St. George’s Respiratory Questionnaire
TDI: Transition dyspnea index
